# Mini-Pterional Craniotomy for Microsurgical Ligation of a Sylvian Fissure Arteriovenous Fistula

**DOI:** 10.7759/cureus.35873

**Published:** 2023-03-07

**Authors:** Rabeeia Parwez, Catherine Zhang, Anthony Ghosh

**Affiliations:** 1 Neurosurgery Department, Queen's Hospital, Romford, GBR

**Keywords:** middle cerebral artery aneurysm, sylvian fissure, venous varix, cognard grading, pial arteriovenous fistula

## Abstract

Pial arteriovenous fistulae (pAVF) are rare lesions, arising from direct fistulation between an artery and vein, with absence of a nidus. We present the surgical treatment of a 22-year-old female found to have a right middle cerebral artery (MCA) pAVF in the Sylvian fissure. The patient underwent a right mini-pterional approach, and careful dissection of the arterial feeder, venous varix and fistulation point. The fistulation point was tested with a temporary clip and intra-operative indocyanine green (ICG) videoangiography. All arterial feeders were identified and ligated. Complete obliteration was confirmed using ICG and post-operative digital subtraction angiography (DSA). The patient made a good recovery with no neurological deficits, and her pulsatile tinnitus stopped. Pial arteriovenous fistulae are rare lesions amenable for a surgical cure in the appropriate context.

## Introduction

Pial arteriovenous fistulae (pAVF) are rare cerebrovascular lesions and account for 1.6% of all intracranial vascular malformations [1,2]. In contrast to arteriovenous malformations, there is an absence of a nidus, and the feeding artery directly fistulates with a draining vein. A number of aetiologies have been proposed to cause pAVF, including congenital, traumatic, and iatrogenic [3]. The most common explanation is congenital, whereby the arteriovenous shunt present during neurovascular genesis fails to regress into capillary beds, leaving a residual direct arteriovenous shunt. Traumatic and iatrogenic causes have also been described, whereby the fistula is caused by direct injury to cortical blood vessels through trauma, or surgical which include craniotomies and bypass procedures.

The Sylvian fissure, also known as the lateral cerebral sulcus, is the most distinct landmark on the lateral surface of the brain. It is formed by the inward folding of the frontal, parietal, and temporal opercula over the insula [4]. The Sylvian fissure extends from the anterior perforated substance to the supramarginal gyrus and the insula forms its floor. It can be further divided into a proximal and distal segment by the anterior Sylvian point (ASP), which in turn leads into the Sylvian cistern where branches of the middle cerebral artery (MCA) lie. The anterior Sylvian point is the most commonly used surgical corridor due to the high proportion of accessible intracranial lesions through here, which includes branches of the MCA. The pterional (frontotemporal) craniotomy is the standard approach to access pathology within the Sylvian fissure and anterior cerebral circulation.

The MCA originates at the bifurcation of the internal carotid artery (ICA), and extends laterally under the anterior perforated space where it reaches and divides in the Sylvian fissure. There are four anatomical segments of the MCA: M1 (sphenoidal or horizontal segment) extends from the terminal ICA bifurcation to the main MCA division. The M2 (insular segment) extends from the main MCA division to the circular sulcus of insula after which the M3 (opercular segment) begins at the circular sulcus of the insula and terminates at the external surface of the Sylvian fissure on the surface of the brain. The M4 (cortical segment) is located on the parasylvian surface of the brain where it extends over the cortical surface of the cerebral hemisphere and terminates.

## Case presentation

A 22-year-old female presented with complaints of otalgia. Further history-taking revealed that she had right-sided pulsatile tinnitus. She was otherwise fit and well with no co-morbidities. Hearing was intact.

**Figure 1:**
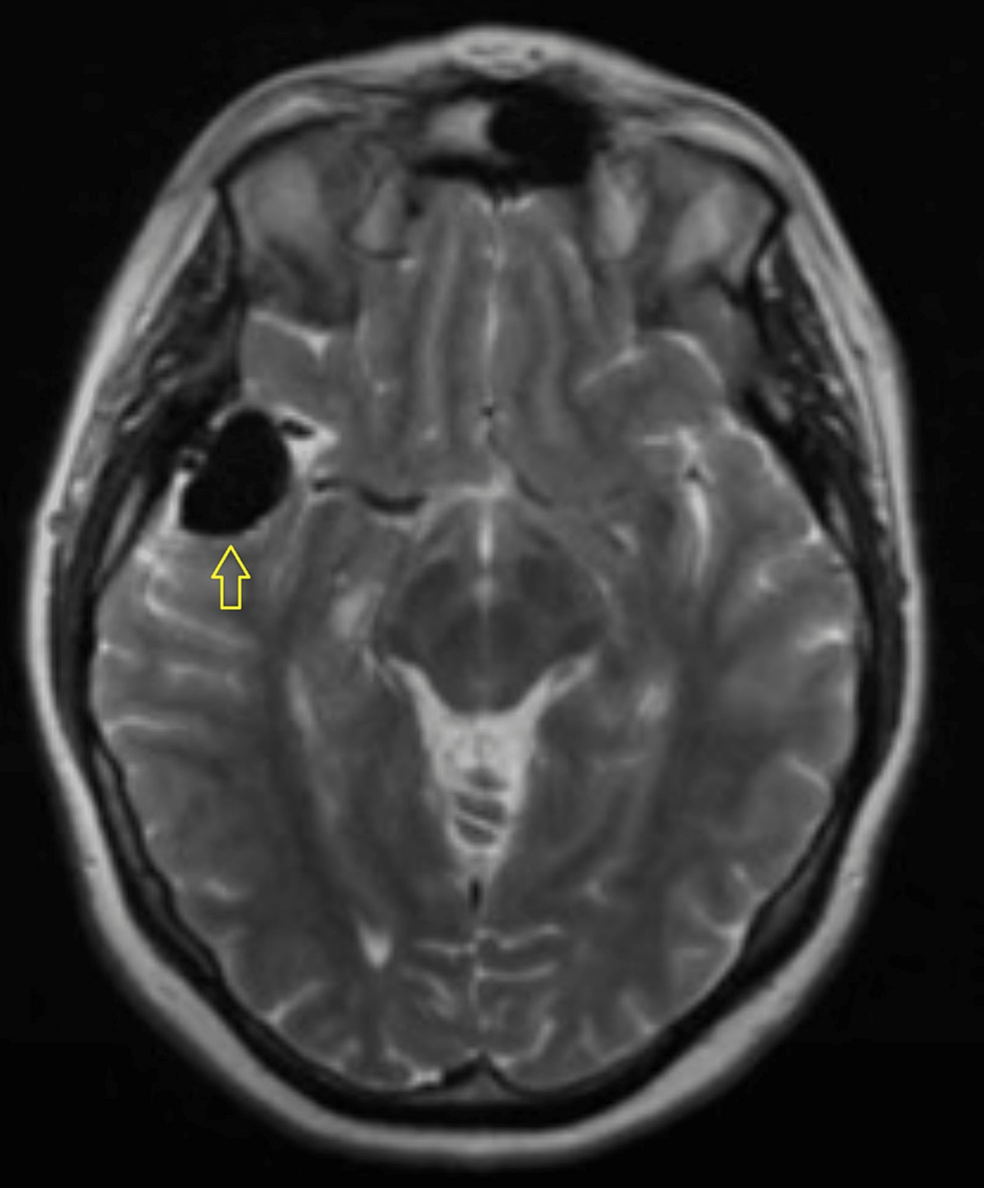
MRI scan showing dilated venous varix in the right Sylvian fissure

**Figure 2:**
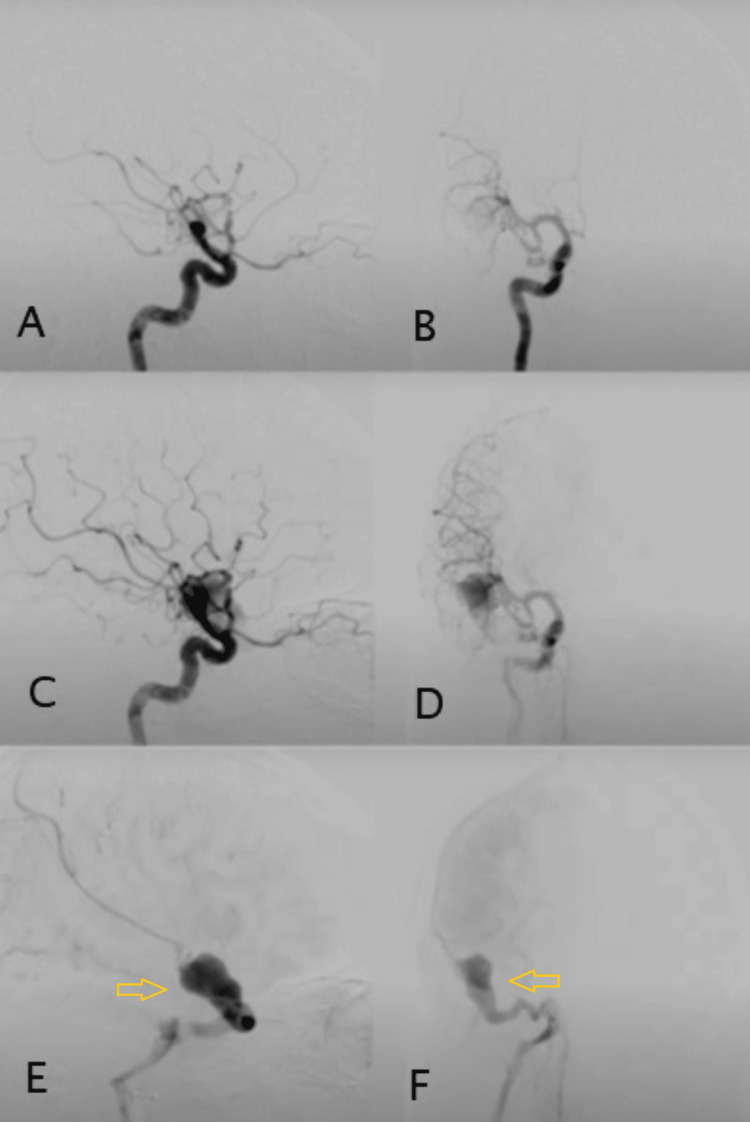
Lateral and anteroposterior (AP) views for a right internal carotid artery injection digital subtraction angiography, demonstrating fast arteriovenous shunting from the right middle cerebral artery into a venous varix which drains into the inferior petrosal vein. Figures 2A-2D show right carotid artery injection digital subtraction angiography Figures 2E, 2F show lateral and anteroposterior views of the venous varix

**Figure 3:**
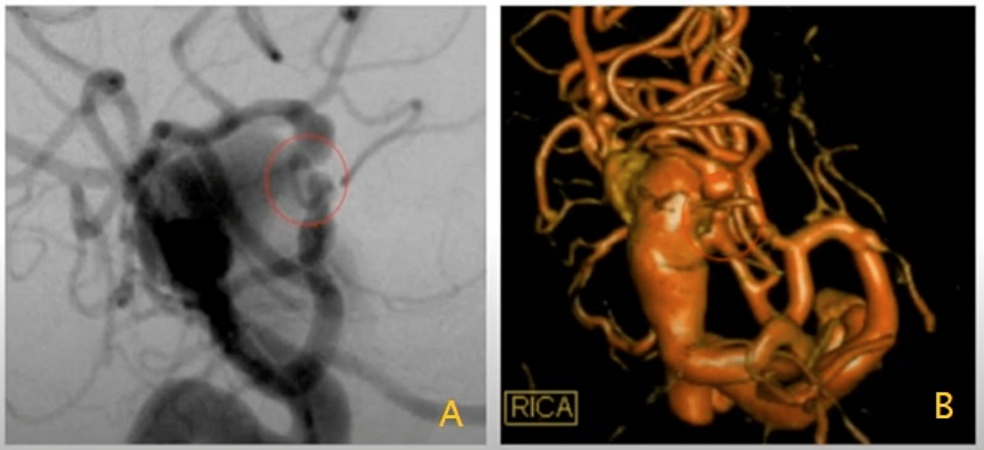
3D reconstruction of the internal carotid artery injection demonstrating the fistulation point Figure 3A demonstrates the fistulation point in digital subtraction angiography Figure 3B demonstrates 3D reconstruction of the fistulation point

MRI head showed a dilated flow void in the right Sylvian fissure (Figure 1). Further digital subtraction angiography (DSA) demonstrated fast arterio-venous shunting, with arterial supply from the right MCA, and venous drainage into the inferior petrosal sinus (Figure 2). There was no associated arteriovenous nidus, and the site of shunting was most likely representative of a pial arteriovenous fistula (Figure 3).

Multidisciplinary team (MDT) discussion adopted the Cognard classification system for dural arteriovenous fistulae to help with risk stratifying this patient, due to the absence of similar classification systems for pial arteriovenous fistulae. This was classified as a Cognard type 4 arteriovenous (AV) fistula, due to the direct drainage into a cortical vein, with an associated venous varix. The annual risk of intracranial haemorrhage would be 8.1%, with a combined annual mortality rate of 10.4%. The options of stereotactic radiosurgery, endovascular treatment, and open microsurgery were considered and discussed with the patient. There were concerns regarding difficulty in finding the point of fistulation to treat using endovascular techniques, and also the high risks of occluding distal MCA branches. Open surgery was therefore deemed to be the most appropriate treatment modality, offering an instant and permanent cure, without high risks of occluding distal MCA branches.

## Discussion

Description of technique

The patient was positioned supine in Mayfield pins, with slight head turn towards left, and malar eminence at the most superior point. Temporalis muscle was preserved with a subperiosteal and subfascial scalp flap, and a mini-craniotomy around the pterion is made with 5mm burr and craniotome. The greater sphenoid ridge and inner table of the skull base were thinned down with a cutting burr.

The Sylvian fissure was split laterally from the anterior Sylvian point, and an M2 branch was identified and exposed proximally to the genu of the MCA thus proximal control was established. Thereafter, following distal dissection of the M2, the large venous varix and its fistulation point with the M2 was exposed.

The fistulation point was then tested with indocyanine green (ICG) videoangiography. A temporary aneurysm clip was applied, and ICG demonstrated perfusion of the MCA branches, but not the varix. After clip removal, the varix was seen to be illuminated and filling with ICG, confirming the point of fistulation. The fistula was coagulated and divided, and repeat ICG videoangiography confirmed no further filling of the varix, with good distal flow to MCA branches.

Presented here is a video with a voice-over description that elaborates the surgical technique employed and a brief history and surgical outcome by Mr. Anthony Ghosh (Video 1).

**Video 1:** Brief case discussion and surgical technique description.

Post-operative DSA confirmed disconnection of the fistula, and no early venous drainage. The patient made a good recovery, with no neurological deficits, and her pre-operative pulsatile tinnitus symptoms had resolved.

Indications

Indications for treatment of pial arteriovenous fistulae are primarily aimed to reduce future risk of haemorrhage. Patients often present with a haemorrhage, seizures, neurological deficits from mass effect from the venous varix, and in rare cases congestive cardiac failure. Indications for surgical treatment are those pAVF that are not amenable to endovascular treatment or stereotactic radiosurgery. The main advantages of surgery over endovascular treatment include lower risk of distal vessel occlusion for certain pAVF, and a higher obliteration rate. Reported obliteration rates in the literature are 96.8% for surgical ligation, compared with 86.5% for endovascular treatments [5]. The main advantage of surgery over stereotactic radiosurgery (SRS) is an instant cure. The potential lifetime risk of haemorrhage with no treatment must also be higher than acceptable risks of surgery.

Limitations

The aim of treatment of pial arteriovenous fistulae is to disconnect the fistulation point, reduce haemorrhage risk, and prevent recurrence. Whilst the lateral trans-Sylvian approach offers good access to lesions in this region, deeper seated lesions can be more challenging to reach and therefore associated with more morbidity. Risks of surgery also include stroke, seizures, infection, cosmetic defects, and risk to life.

How to avoid complications

Complications can be minimized with good surgical planning, prior MDT discussion and appropriate patient counselling. Establishing early proximal control prior to dissecting the fistula and varix can minimize catastrophic intra-operative bleeding. Common bleeding points include the fistulous point and venous varix, and careful dissection should also be performed around these regions.

With a mini-pterional approach to access the Sylvian fissure, a subperiosteal and subfascial flap can reduce risk of injury to the frontal branch of the facial nerve, and subsequent facial muscle weakness. It also can potentially reduce temporalis muscle wasting due to minimal electrocautery of the muscle.

Cosmetic dissatisfaction with a pterional craniotomy can also be reduced by good alignment of the bone flap in the superoanterior aspect of the craniotomy, and resecuring the deep temporalis fascia to the superior temporal line on closing.

Specific information for the patient

Our patient presented with pulsatile tinnitus, most likely due to the audible turbulent blood flow through an abnormal arteriovenous shunt [5]. Compared with the literature, most common presentation of pAVF in adults is haemorrhage, followed by mass effect and seizures [5]. Risks of bleeding can be difficult to predict in similar cases, due to the rarity of this condition. However these lesions are often considered similar to Cognard type 4, or Borden type III, with reports suggesting an unfavourable natural history and up to 63% mortality. Most case series in the literature therefore advocate treatment for pAVF. Interestingly, the presence of venous varices in pAVF has been described to correlate with a lower risk of intracranial haemorrhage, with the postulation that a dilated varix acts as a pressure buffer [6,3].

Whether the venous varix should be excised during surgery is still debatable [1]. Excision of the entire lesion (varix and associated arterial feeders) has been performed in the past, when pAVF were regarded as a form of arteriovenous malformation, in order to prevent recurrence. Whilst this may still be necessary for pAVF with multiple arterial feeders, recent reports suggest that complete disconnection of the shunt (either surgically or endovascularly) without excision of the varix is sufficient [6]. From our experience with this patient, coagulation and ligation of the single fistula point was a sufficient cure with no recurrence.

## Conclusions

Pial AVFs are rare lesions and management requires MDT decision-making. Patients must be appropriately counselled on operative risks versus risks of future bleeding. Pulsatile tinnitus can be a symptom of intracranial arteriovenous shunting due to the turbulent blood flow, and complete obliteration of the shunt can cure the pulsatile tinnitus. Pial AVFs in the Sylvian fissure are readily accessible through a standard pterional craniotomy. Small craniotomy, subperiosteal and subfascial flap can be performed to minimize cosmetic defects. Caution must be taken during dissection of the venous varix and the fistulation point, which carry the highest risks of bleeding. Ensure all arterial feeders are identified and ligated and then test the fistulation point with a temporary clip. It is important to use intra-operative ICG to confirm obliteration of the fistula, and follow up with a DSA. The need to resect the venous varix is still debatable. In our case study, coagulation and ligation of a single lumen pial arteriovenous fistula without excision of the varix was sufficient to cure the patient with no subsequent recurrence.
